# Germline variant in *REXO2* is a novel candidate gene in familial pheochromocytoma

**DOI:** 10.1017/S0016672320000038

**Published:** 2020-05-01

**Authors:** Yael Laitman, Shay Tzur, Ruben Attai, Amit Tirosh, Eitan Friedman

**Affiliations:** 1 The Susanne Levy Gertner Oncogenetics Unit, The Danek Gertner Institute of Human Genetics, Tel HaShomer, Israel; 2 Genomic Research Department, Emedgene Technologies, Tel Aviv, Israel; 3 Institute of Endocrinology, Unit of Neuroendocrine Tumors, Sheba Medical Center, Tel HaShomer, Israel; 4 The Sackler School of Medicine, Tel Aviv University, Tel Aviv, Israel

**Keywords:** inherited predisposition, pheochromocytoma, *REXO2* gene, whole-exome sequencing

## Abstract

Pheochromocytoma (PCC) is a rare, mostly benign tumour of the adrenal medulla. Hereditary PCC accounts for ~35% of cases and has been associated with germline mutations in several cancer susceptibility genes (e.g., *KIF1B*, *SDHB*, *VHL*, *SDHD*, *RET*). We performed whole-exome sequencing in a family with four PCC-affected patients in two consecutive generations and identified a potential novel candidate pathogenic variant in the *REXO2* gene that affects splicing (c.531-1G>T (NM 015523.3)), which co-segregated with the phenotype in the family. *REXO2* encodes for RNA exonuclease 2 protein and localizes to 11q23, a chromosomal region displaying allelic imbalance in PCC. REXO2 protein has been associated with DNA repair, replication and recombination processes and thus its inactivation may contribute to tumorigenesis. While the study suggests that this novel *REXO2* gene variant underlies PCC in this family, additional functional studies are required in order to establish the putative role of the *REXO2* gene in PCC predisposition.

## 1. Introduction

Pheochromocytoma (PCC; OMIM #171300) is a rare neuroendocrine tumour of the adrenal medulla. The most prevalent symptoms associated with this tumour, also known as the ‘PCC triad’, are severe headaches, palpitations and excessive sweating, which are typically episodic (Bryant *et al.*, 2003). PCC is responsible for up to 0.1% of new cases of hypertension, episodic or sustained (Pacak, 2001). PCCs are mostly benign tumours that exhibit variable prevalence rates across populations. The estimated prevalence of PCC is between 1:6500 and 1:2500, with the annual incidence of 500–1600 cases per year in the USA (Chen *et al.*, 2010). The incidence of PCC in The Netherlands has reportedly increased from 0.29 per 100,000 in 1995–1999 to 0.46 per 100,000 from 2011 to 2015 (Berends *et al.*, 2018). There are no data available on PCC occurrence rates in Israel. As is the prevailing paradigm for all tumours, PCC development is associated with and driven by the accumulation of somatic mutations (Burnichon *et al.*, 2011). Inherited predisposition to PCC, implying germline mutations that increase PCC risk, are detected in ~35% of incident PCC cases (Neumann *et al.*, 2002, 2018). Some inherited PCC cases are encountered in the context of specific cancer susceptibility syndromes, such as multiple endocrine neoplasia type 2A and B (OMIM #171400 and #162300, respectively), von Hippel–Lindau syndrome (OMIM #193300) and neurofibromatosis type 1 (OMIM #162200). In nearly 60% of all *hereditary* PCC cases, driver germline mutations have been detected in up to 20 genes, including *MAX*, *NF1*, *RET*, *SDHA*, *SDHB*, *SDHC*, *SDHD*, *TMEM127*, *FH*, *VHL* and others (Rattenberry *et al.*, 2013, Martucci & Pacak, 2014, Buffet *et al.*, 2018).

As PCC is a rare disease, it is recommended that all cases of PCC undergo genetic counselling and genotyping for the possibility of identifying germline variants in the known PCC susceptibility genes, irrespective of age at diagnosis and/or family history of endocrine tumours that may be suggestive of an inherited predisposition (Muth *et al.*, 2019). While a substantial proportion of familial PCCs are associated with germline mutations in any of the known PCC susceptibility genes, in a subset of families with an unusual clustering of PCC cases, the genetic basis for this clustering remains elusive. In the present study, we applied whole-exome sequencing (WES) in order to define germline pathogenic sequence variants (PSVs) in an Israeli family where PCC was diagnosed in four relatives across two successive generations.

## 2. Materials and methods

### 2.1. Clinical study

**Fig. 1.**
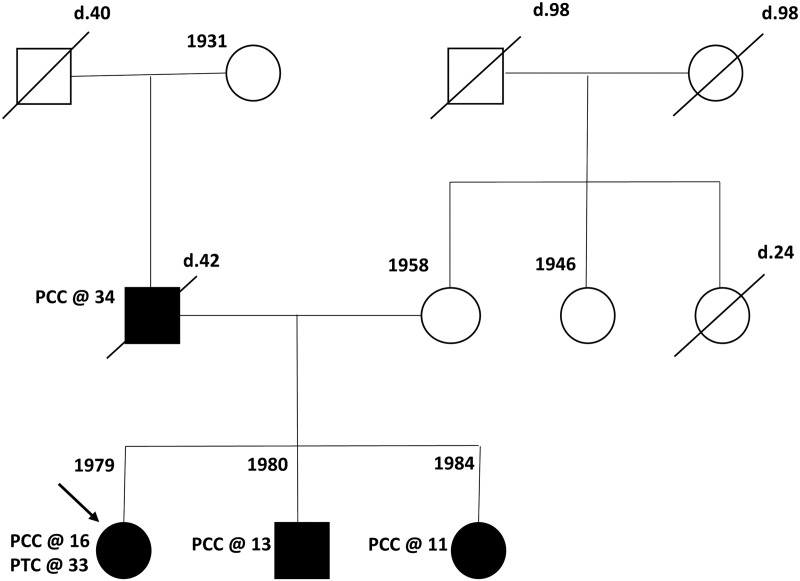
Pedigree. The affected individuals are shaded, age at diagnosis of tumours is also denoted and year of birth is shown next to the depicted individual. PCC = pheochromocytoma; PTC = papillary thyroid cancer.

The proband, a woman born in 1978 to a Bulgarian Jewish mother and a non-Jewish Bulgarian father, was referred for oncogenetic counselling at the Oncogenetics Unit, Sheba Medical Center, in October 2015. She was diagnosed with PCC at age 15 after a year of symptoms including recurrent palpitations, pre-syncopal attacks and episodic hypertension (up to 210/110 mmHg). She underwent unilateral adrenalectomy at age 16 (in Bulgaria) for the diagnosis of PCC. At age 33, she was diagnosed with papillary thyroid cancer, underwent surgical resection and was treated with radioactive iodine. At her last follow-up (April 2019), she was asymptomatic, euthyroid on thyroid replacement therapy, normotensive and with no evidence of PCC based on biochemical analysis of urine catecholamine and serum metanephrine levels and abdominal computed tomography (CT) imaging. Her family history was noted for a father who was diagnosed with malignant PCC at age 32 and died of metastases at age 44 (in Bulgaria) and an unaffected mother (of Bulgarian Jewish ancestry). The proband's younger sister and brother were both diagnosed with unilateral PCC at age 13 and 11, respectively. Their diagnosis was prompted by the diagnosis of PCC in their father. Both siblings were operated on in Bulgaria, and at the time of reporting (June 2019) were asymptomatic with no evidence of disease. Notably, clinical and radiological evaluation (including physical examination, ophthalmological examination, abdominal ultrasound and CT and central nervous system magnetic resonance imaging) performed during 2019 as part of the clinical follow-up in the proband was negative. Figure 1 depicts the pedigree and relevant tumours and ages of affected members at diagnosis.

The experimental protocols for this study were approved by the Institutional Review Board at the Sheba Medical Center. All participants provided written informed consent before undergoing the genetic tests.

### 2.2. Genetic analyses

Peripheral blood DNA was extracted from the two affected female individuals, their affected male sibling and the unaffected mother using the Qiagen QIAmp DNA extraction kit (Germantown, MD, USA) following the manufacturer's recommended protocol. Initially, polymerase chain reaction (PCR)-amplified, exon-centred Sanger sequencing was applied in order to look for the presence of germline mutations in the *VHL* and *RET* genes in the proband. Subsequently, WES using the Roche NimbleGen V2 chip (Madison, WI, USA) with the Illumina HiSeq2000 sequencing platform was performed on DNA extracted from both affected sisters and their unaffected mother. Later, Sanger sequencing was used to validate the variants identified through WES in all available family members (see below).

### 2.3. Reverse transcription PCR

Leukocyte RNA isolation and purification were carried out using the ‘NucleoSpin RNA Blood’ kit manufactured by Macherey-Nagel (Fisher Scientific, Leicestershire, UK). For generation of cDNA, we used the ‘qscript cDNA synthesis’ kit by QuantaBio (Beverley, MA, US). Primers used for amplification were:

REXO2RT_F: GTAGGTGGGAGTCACGGACG

REXO2RT_R: CGGTAAAACTGAAGCTCTTTGATGC

Following 30 amplification cycles, the PCR products were separated on 3% agarose gel and visualized with ethidium bromide staining.

### 2.4. Variant calling and annotation

For each of the WES-genotyped samples, raw data were uploaded to Emedgene's Health Insurance Portability and Accountability Act (HIPAA)-compliant genomic platform ‘WELLS’, which includes an internal bioinformatic pipeline for genomic analysis, and ‘ADA’, which includes automatic interpretation algorithms (www.emedgene.com). The reads were mapped against the human reference genome hg19/GRCh37 using the BWA MEM algorithm (Pabinger *et al.*, 2014; Jager *et al.*, 2016). A variant call format file was generated for each sample using multiple sets of variant callers (including GATK4, SAMTOOLS and FREEBAYES) (Li *et al.*, 2009, McKenna *et al.*, 2010, Guo *et al.*, 2015). All shortlisted candidate variants were annotated using Variant Effect Predictor. Ultra-rare variants (<0.01% in gnomAD) that followed an autosomal dominant inheritance mode in affected samples and exhibited a clear deleterious effect on the transcript were further investigated. In order to assess the potential pathogenicity and relevance of these candidate variants to PCC predisposition, a series of additional features were considered, including: (1) expression in neuroendocrine tissue; (2) localization to chromosomal regions that display somatic allelic imbalances in PCC; (3) a phenotype that is not mutually exclusive with a role in familial PCC (e.g., multiple skeletal defects, intellectual disability); and (4) matching family segregation of the alternative allele using Sanger sequencing.

## 3. Results

### 3.1. Sanger sequencing of the major candidates genes

Sanger sequencing was applied in order to assess the presence of *VHL* and *RET* gene pathogenic variants initially in the proband and subsequently in both affected siblings. No pathogenic or likely PSVs in these two genes were detected in any of the affected family members.

### 3.2. WES analysis and candidate genes

The exomes of the two affected sisters and their unaffected mother were sequenced, and 24,119 variants in coding regions were found in the family. Initially, analysis of the genotypes of the known PCC-related genes was carried out in both affected sisters (*MAX*, *NF1*, *RET*, *SDHA*, *SDHB*, *SDHC*, *SDHD*, *TMEM127*, *FH* and *VHL*). In addition, the sequencing coverage of the coding regions of these genes was manually inspected in order to ensure that regions with low-level coverage did not contain additional suspected variants (all coding regions of these genes represented >20× coverage of at least one of the affected family members). Sequencing data from both affected sisters exhibited no pathogenic or likely pathogenic variants in any of the known genes related to PCC predisposition. Yet, it is still possible that the coverage was insufficient to detect existing mutations or that there were major gene rearrangements affecting any of these known PCC predisposition genes that were not detected herein. At least for the *RET* and *VHL* genes, this seems less plausible as heterozygous sequence variants (single-nucleotide polymorphisms) were detected within these genes in at least one affected individual.

Seemingly irrelevant sequence variants were filtered out based on allele frequency and effect (as outlined above), as well as family segregation based on the expected zygosity (under an autosomal dominant mode of inheritance). Originally, 24,119 exome variants were found in the family. These variants were filtered according to the following process: variants that are segregated with the disease (heterozygote zygosity in all three affected siblings and reference allele in mother; 3438 variants remain); variants with allele frequencies of <1% in public databases and local databases and also with an allele count of <20 in gnomAD (61 variants remain); and variants with predicted loss of function (LoF; 4 variants remain). The four LoF variants were further validated using Sanger sequencing in all three affected siblings and defined as the potential candidates. The four validated gene variants shortlisted for further consideration are discussed in the following.

A heterozygous, stop-gain variant (NM_001037324.2:c.1561C>T- NP_055508.3:p.Arg668Ter, chr3:184008139(hg19)) was found in the *ECE2* gene (OMIM #610145) that co-segregated with the phenotype. However, its limited tissue expression (primarily in the liver, ovary, prostate, myometrium and thrombocytes) with no discernible expression in the adrenal gland (https://varsome.com/gene/ECE2; https://www.proteomicsdb.org/proteomicsdb/#human/proteinDetails/49371/expression), makes it an unlikely candidate. In addition, this gene is relatively tolerant to LoF variants: a total of 255 individuals are listed with LoF in gnomAD database (https://gnomad.broadinstitute.org/gene/ENSG00000145194) (pLi score = 0.00). The p.Arg668Ter variant has been reported 18 times on gnomAD, and infrequently among Asian (0.0004, 8/19,948) and European (0.0001, 8/129,022) populations. The frequency of LoF variants in this gene (~120/280,000) is much higher than disease prevalence (~0.5 per 100,000). These factors combined have led to the consideration that the *ECE2* gene variant is an unlikely contributor to PCC predisposition in this family.

A heterozygote variant in the *TTC39A* gene, a variant located within the start-codon of one of the gene transcripts (NM 001297664.1: c.1A>G; chr1:51787457 (hg19)) was also considered. This variant is a missense variant in the other transcripts of this gene (p.Met32Val, p.Met28Val). Despite the putative predicted deleterious effect of this variant as a start-loss on protein formation, it only affects one of the transcripts of this gene. In addition, several other factors have led to its low priority as a PCC susceptibility candidate: low expression in adrenal glands and the additional phenotypic features known to be associated with pathogenic mutations in this gene (e.g., lipid metabolism and cardiovascular disease; https://www.ncbi.nlm.nih.gov/gap/phegeni?tab=1&gene=22996). In addition, the gene has moderate tolerance to LoF mutations (pLi = 0.10; gnomAD reports 49 high-quality LoF variants in this gene, which is also higher than the expected frequency compared with disease rates).

**Fig. 2.**
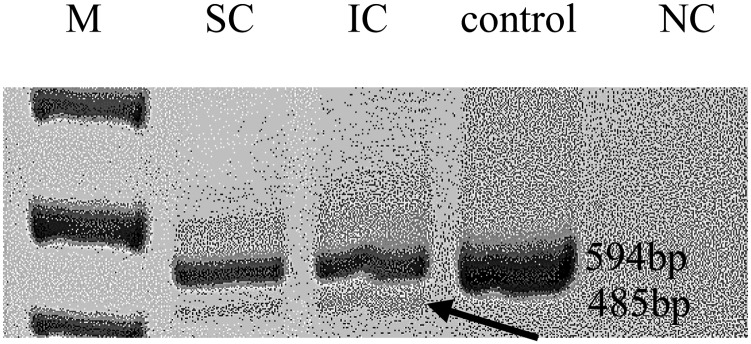
Reverse transcription polymerase chain reaction. Arrows mark the predicted abnormal splice transcript. M = size marker; SC/IC = *REXO2* carriers; NC = negative control.

The most intriguing variant was a splice-site variant found in the RNA exonuclease 2 (*REXO2*; OMIM #607149) gene at position c.531-1G>T (NM_015523.3, chr11:114318547G>T). The alternative allele of this variant was detected in all three affected individuals in the studied family. *REXO2* localizes to the 11q23 area, a chromosomal region exhibiting allelic loss in human PCC (Sun *et al.*, 2006). The variant is located within the conserved splicing site and seems to have a predicted deleterious effect on splicing by multiple prediction tools (TraP score = 0.584, spliceAI = 0.89) (Gelfman *et al.*, 2017, Jaganathan *et al.*, 2019). In addition, reverse transcription PCR on RNA products confirmed this mutation as affecting the splicing of that gene (Figure 2). Remarkably, the gnomAD database includes only 22 classified LoF variants in this gene. Notably, few other variants that predictably affect the same splice site (TraP score >0.2) were identified in the gnomAD database: c.531A>G and c.531-4A>G in one sample each and c.531-6G>A and c.531-6G>T in three and two samples, respectively.

The *REXO2* gene is ubiquitously expressed in multiple tissues, including the adrenal glands (https://www.genecards.org/cgi-bin/carddisp.pl?gene=REXO2&keywords=rexo2&prefilter=interactions#interactions). This gene encodes for an enzyme that is present in both the cytosol and mitochondria. Nonetheless, loss of the functional protein is reported to affect the mitochondrial environment more severely than compared to the cytoplasmic matrix (Bruni *et al.*, 2013). The splice mutation can also affect mRNA transcripts and inhibit or alter some metabolic enzymes that are essential for cell viability and growth (Bruni *et al.*, 2013). The *REXO2* gene and the specific variant identified in the current study have not been reported as a PCC-associated gene in earlier studies (Rattenberry *et al.*, 2013, Fishbein *et al.*, 2017; Buffet *et al.*, 2018).

## 4. Discussion

In this study, a potential PSV in the *REXO2* gene was identified as a novel candidate for PCC predisposition. The considerations that have led to assigning a putative pathogenic and causative role to this gene variant in PCC predisposition are: it co-segregates with the phenotype in the studied family; it does not exist in publicly available databases; mutations in this gene are rare in the general population; the tissue expression pattern of the gene product; its chromosomal location; and the likely effect of this mutation on gene splicing. Clearly, the previously reported genes that have been associated with inherited PCC were excluded as contributing factors to the phenotype in the family reported herein.

*REXO2* is the member of the DEDD superfamily mediating various steps of RNA and DNA processing (Yang *et al.*, 2011; Chu *et al.*, 2019) and is pivotal in the degradation of nanoRNAs (Zhang *et al.*, 1998). The REXO2 protein can also degrade small DNAs, suggesting a role in cellular and mitochondrial deoxynucleotide recycling (Nguyen *et al.* 2000; Bruni *et al.* 2013). Reduced concentration of REXO2 in HeLa cells can severely impair mitochondrial morphology and cell growth (Bruni *et al.*, 2013). In addition, mitochondrial protein synthesis is also decreased in the absence of the REXO2 enzyme, which may further disrupt protein translation by altering mtDNA replication, repair or recombination processes (Bruni *et al.*, 2013). These studies show that REXO2 plays an essential role in DNA and RNA metabolism, and a lack of this enzyme may lead to abnormal double-strand breaks and failure of DNA repair and recombination processes, leading to cell-cycle arrest and abnormal apoptosis (Bruni *et al.*, 2013).

A somatic mutation database reports 45 unique *REXO2* mutations in a total of 47,818 tumour samples (https://cancer.sanger.ac.uk/cosmic/search?q=REXO2#), and 14 LoF germline *REXO2* mutations have been reported in gnomAD (https://gnomad.broadinstitute.org/gene/ENSG00000076043).

Loss of heterozygosity at 11q23, the location of the *REXO2* gene, has been described in PCC (Sun *et al.*, 2006), a known mechanism that may serve to inactivate the wild-type allele and may contribute to PCC pathogenesis. Not having access to the adrenal tumours resected in this family, such additional supporting evidence for the role that REXO2 may play in PCC pathogenesis could not be provided in the present study.

The exact mechanisms by which *REXO2* may confer PCC predisposition are not clear. It seems plausible that the LoF mutation leads to a dysfunctional REXO2 enzyme, resulting in disruption of DNA repair mechanisms (Hsiao *et al.*, 2014) and abnormal ribosome biogenesis that can arrest the cell cycle in the growth phase (Gomez-Herreros *et al.*, 2013; Polymenis & Aramayo, 2015; Shamsuzzaman *et al.*, 2017). Impairment of ribosome biogenesis has also been linked with the activation of tumour suppressor genes, such as p53 (Hölzel *et al.*, 2010; Golomb, 2014), which can eventually result in various tumorigenesis processes. REXO2 is also likely to serve a preventive role in UV-C-induced cell death, and its absence can induce detrimental effects on cellular and metabolic processes (Ito *et al.*, 2004).

Obviously, this is the first report to suggest that *REXO2* gene variants might be involved in PCC predisposition. However, firm conclusions cannot be drawn from this single family: the lack of available tumour tissue to test for allelic loss or immunostaining for loss of protein expression, the lack of an animal model to conclusively show the causal role of this gene variant in PCC, the existence of other *REXO2* variants affecting the same splice site and the fact that no other PCC families were reported to harbour mutations in this gene should be taken into consideration. Clearly, future research should focus on validation of the putative pathogenic role of this gene in PCC predisposition and tumorigenesis using knockout experiments, *in vitro* cell line experiments and the genotyping of more PCC families for *REXO2* mutations.

In conclusion, a *REXO2* germline mutation may contribute to familial-inherited PCC, yet the evidence to support this claim is inconclusive at this stage. The contribution of this gene to other familial PCC families as well as the exact pathogenic mechanism require further studies.
